# Levothyroxine-induced liver injury followed by complete recovery upon cessation of the drug: a case report

**DOI:** 10.1186/s13256-019-2244-z

**Published:** 2019-10-18

**Authors:** Abbas F. Hlaihel, Mudher Z. H. Al-Khairalla

**Affiliations:** 1 ThiQar Medical School, Nasiriyyah, Iraq; 2 ThiQar Lung Diseases Centre, Nasiriyyah, Iraq

**Keywords:** Case report, Levothyroxine, Hepatitis, Hypothyroidism, Triiodothyronine

## Abstract

**Background:**

Levothyroxine is a synthetic thyroxine and is the treatment of choice for hypothyroidism. It is a prohormone with minimal intrinsic activity. The drug is de-iodinated in peripheral tissue to form triiodothyronine, which is the active thyroid hormone. On initiation of treatment, levothyroxine is titrated, and usually it is extremely well tolerated in the vast majority of patients. We report a case of a patient with self-limiting levothyroxine-induced liver injury, a rare adverse effect of this drug.

**Case presentation:**

We report a case of a 34-year-old Mediterranean woman diagnosed with post-thyroidectomy hypothyroidism. She was commenced on levothyroxine and developed liver injury confirmed by noninvasive liver investigations. Complete recovery of the patient’s liver tests occurred upon cessation of the drug. Triiodothyronine was an appropriate treatment alternative.

**Conclusion:**

Levothyroxine-induced liver injury is a rare, and in the present case report, a self-limiting, adverse effect. The diagnosis of our patient was confirmed via noninvasive diagnostic methods. Knowledge of this rare adverse effect is important in the differential diagnosis of patients who have commenced on levothyroxine and have deranged liver enzymes in the context of hypothyroidism.

## Introduction

Normal thyroid function is crucial for homeostasis. In most patients diagnosed with hypothyroidism, treatment in the form of thyroid replacement is lifelong. Levothyroxine is the commonest drug used in the treatment of hypothyroidism and is preferred over triiodothyronine because its prohormone nature allows the patient’s own physiological mechanisms to control the conversion into its active form. Levothyroxine is widely available, and it is extremely well tolerated by most patients. The dose is titrated according to clinical response and thyroid function assay.

An exceedingly rare side effect of this drug is induced liver injury. This has rarely been reported in the literature [1–3]. In our reported case, cessation of levothyroxine led to complete resolution of the drug-induced liver injury (DILI). This case report supports the premise that in some selected cases of DILI, a liver biopsy is not required to confirm the diagnosis. This is a useful addition to existing medical literature to help guide management pathways in cases of suspected DILI.

## Case presentation

We report a case of a 34-year-old Mediterranean woman living in a rural area in Thi-Qar Province in southern Iraq. She presented to our hospital with upper abdominal pain, anorexia, and nausea. She also had a 1-week history of low-grade fever and fatigue. She had no other symptoms upon general systematic inquiry; in particular, she had no history of gastrointestinal symptoms, no joint pain symptoms, and no photosensitivity or history of abortions. She had a regular menstrual cycle. Two weeks prior to her presentation, she underwent a thyroidectomy for a suspicious thyroid nodule. The histology of the nodule was confirmed as benign. She was not exposed to halothane during general anesthesia. She had been commenced on 50 μg of levothyroxine 10 days prior to this presentation.

She had no other past medical history of note and had received no previous prescribed medication. In particular, she had no history of blood transfusion, liver disease, or alcohol consumption. She did not report a family history of liver disease. She had never smoked, and she lived with her husband and young children at home. She was a stay-at-home mother and held no other occupation. On examination, she had normal vital signs: Her pulse rate was regular at 68 beats per minute with normal volume, respiratory rate was 10 breaths per minute, temperature was 36.8 °C, and blood pressure was 108/63 mmHg. Pulse oximetry revealed saturation of 96% on room air at rest. Her physical examination did not reveal any jaundice, and she had no stigmata of chronic liver disease. Examination of her cardiorespiratory systems revealed pure heart sounds with no murmur, and her respiratory auscultation revealed vesicular breath sounds and central trachea with no localizing signs or added sounds. A complete neurological examination confirmed a normal Mini Mental State Examination score, and she was oriented to time, place, and person. Her cranial nerves were intact, and the tone, power, reflexes, and sensory examination of the upper and lower limbs were symmetrically normal. Investigations confirmed acute hepatitis with raised urinary bilirubin, raised serum bilirubin 1.4 mg/dl, alanine aminotransferase (ALT) 549 U/L, aspartate aminotransferase (AST) 372 U/L, and alkaline phosphatase (AP) 338 U/L (see Table 1). A gamma-glutamyl transferase measurement was not available at the time of presentation. The result of the patient’s abdominal ultrasonography was normal.

**Table 1:** Laboratory test results

Date*	March	*March 12*	March 14	March 15	March 23	*March 26*	March 27	March 31	**April 4**	April 5	April 9	April 15	April 18	April 20	April 24	May 1	May 16	September 12	Units
TSH				12.7	32.6				>75.0							34.8	21.06		0.27–4.20 IU/L
fT4																	10.58		pmol/L
T4									40.81							30.65			66–181 nmol/L
T3									0.72							2.9	2.52		1.2–3.1 nmol/L
D.Bil	2.4	1.4			1.26	1.1		0.7		0.5	0.5	1.4	0.4		0.4				<0.5 mg/dl
T.Bil					1.73	2.0		1.3		1.3	1.5		0.8	0.8	1.0			0.5	0.2–1.2 mg/dl
AST		372			26	195		74		49	45		33	25	31			15	5–34 U/L
ALT		549			69	486		191		101	86		56	35	41			18	0–55 U/L
AP		383			97	153		120		87	94		77		65				40–150 U/L
S.Alb		28				3.3								3.9				4.1	3.5–5.0 g/dl
HepBsAg		Negative																	
Anti-HCV		Negative																	
Anti-HAV IgG		Negative																	
Anti-HAV IgM		Negative																	
Anti-HEV IgM		Negative																	
Anti-CMV IgG		Positive																	
Anti-CMV IgM		Negative																	
Anti-LKM-1 Ab			1.8																<10 U/ml
ASMA			1.9																<15 IU/ml
ANA			0.3																<1 IU/ml
Ceruloplasmin			45.94																22–61 mg/dl
Hb							11.9												g/dl
WBC							8.2												×10^9^/L
Plt							267												×10^9^/L

*Abbreviations: ALT* Alanine aminotransferase, *ANA* Antinuclear antibody, *Anti HAV* Anti-hepatitis A virus antibody, *Anti HCV* Anti-hepatitis C virus antibody, *Anti HEV* Anti-hepatitis E virus antibody, *Anti-LKM − 1 Ab* Liver kidney microsome type 1 antibody, *AP* Alkaline phosphatase, *ASMA* Anti-smooth muscle antibody, *AST* Aspartate aminotransferase, *CMV* Cytomegalovirus, *D. Bil* Serum direct bilirubin, *fT4* Free thyroxine, *Hb* Hemoglobin, *HepBsAg* Hepatitis B surface antigen, *Plt* Platelets, *S. Alb* Serum albumin, *T. Bil* Total serum bilirubin, *T3* Triiodothyronine, *T4* Thyroxine, *TSH* Thyroid-stimulating hormone, *WBC* White blood cell count

*Dates are all in 2018. Those in Italic font coincide with cessation of levothyroxine (second date was after the patient was rechallenged). Dates in Bold font coincided with commencement of T3 treatment

The differential diagnoses were; viral Hepatitis, drug induced autoimmune hepatitis, drug induced hepatitis, non-alcoholic steatohepatitis (NASH) and sepsis with ‘bystander’ hepatitis

She had a negative virology screen (anti-hepatitis A virus antibody immunoglobulin M [IgM]-negative, hepatitis B surface antigen-negative, anti-hepatitis C virus-negative, anti-hepatitis E virus IgM-negative, anti-cytomegalovirus IgM-negative). Results of an autoantibody screen, including antinuclear antibody, anti-smooth muscle antibody, and anti-liver kidney microsome type 1 antibody, were negative. Serum ceruloplasmin was normal at 46 g/dl. We suspected levothyroxine-induced acute hepatitis and asked the patient to discontinue her medication. No baseline liver tests were available before the introduction of levothyroxine. Within 1 week, the patient had a dramatic response clinically. Biochemically, her liver enzymes (ALT, AST, and AP) and serum bilirubin normalized. The patient consulted another clinician privately, who attempted low-dose rechallenge with levothyroxine, and this led to recurrence of her liver enzyme derangement, which again subsequently subsided upon withdrawal of the drug. The patient’s hypothyroidism was treated with triiodothyronine 35 μg/day with subsequent clinical and biochemical improvement, and no evidence of recurrence of liver enzyme derangement was observed.

The patient was treated by cessation of the levothyroxine, which led to normalization of her liver enzymes. Her hypothyroidism was treated with triiodothyronine instead. The patient’s clinical progress and her thyroid and liver function tests were kept under surveillance over the next 6 months. Her thyroid function responded favorably to the introduction of triiodothyronine, and her liver enzymes completely normalized within 4 weeks after discontinuation of levothyroxine and remained normal until her 6-month follow-up appointment.

## Discussion

We report a case of a 34-year-old woman who developed post-thyroidectomy hypothyroidism requiring thyroid replacement. She was initially treated with levothyroxine; however this led to DILI, which resolved upon cessation of the drug. The patient’s hypothyroidism was managed with triiodothyronine, and she responded favorably. This case report adds to the medical literature in two important points. First, levothyroxine can rarely lead to liver injury, which is a significant adverse effect about which clinicians should be vigilant. Second, no invasive liver biopsy was required to support the diagnosis in this particular case, highlighting that a less invasive approach in DILI can occasionally suffice in securing the diagnosis.

DILI has been implicated in association with over 1000 drugs and herbal products. DILI can be classified in several ways. Initially, classification is based on clinical presentation (hepatocellular, cholestatic, or mixed injury). Occasionally, a liver biopsy is required to make the diagnosis and assess the extent of damage. Histological findings include hepatitis, cholestasis, and steatosis. A liver biopsy was not obtained in this case, because results of testing for an alternative cause were negative, and the patient’s liver enzymes normalized immediately after the drug was discontinued. In general, requesting liver enzyme tests at baseline and for monitoring purposes is reserved for common culprits such as isoniazid and methotrexate. Levothyroxine-induced liver injury is an exceedingly rare adverse effect. In our patient’s case, we attributed liver injury to this drug on the basis of the facts that drug exposure preceded the onset of liver injury, underlying liver disease was excluded, cessation of the drug led to improvement in liver enzymes, and symptoms recurred rapidly when the patient was rechallenged. Indeed, a total score of 8 on the Naranjo Adverse Drug Reaction Probability Scale (Table 2) was observed, supporting a causal effect. The Roussel Uclaf Causality Assessment Method diagnostic score was 11, suggesting that an adverse drug reaction was highly probable. The improvement in liver enzymes upon cessation of the drug also argues against transaminitis due to the hypothyroidism itself. Sepsis with “bystander” hepatitis is a reasonable differential diagnosis to make because the patient did present with abdominal pain and fever. However, we considered it less likely in view of the transaminitis that occurred upon rechallenge of levothyroxine when the patient was clinically well and her liver enzymes had begun to normalize.

**Table 2:** Naranjo Adverse Drug Reaction Probability Scale scoring system

Question	Yes	No	Do not know	Score
Are there previous conclusive reports on this reaction?	+1	0	0	+1
Did the adverse event appear after the suspected drug was administered?	+2	−1	0	+2
Did the adverse reaction improve when the drug was discontinued or a specific antagonist was administered?	+1	0	0	+1
Did the adverse event reappear when the drug was readministered?	+2	−1	0	+2
Are there alternative causes (other than the drug) that could on their own have caused the reaction?	−1	+2	0	+2
Did the reaction reappear when a placebo was given?	−1	+1	0	0
Was the drug detected in blood (or other fluids) in concentrations known to be toxic?	+1	0	0	0
Was the reaction more severe when the dose was increased or less severe when the dose was decreased?	+1	0	0	0
Did the patient have a similar reaction to the same or similar drugs in any previous exposure?	+1	0	0	0
Was the adverse event confirmed by any objective evidence?	+1	0	0	0
Total score				8

The patient had a disproportionate elevation in serum aminotransferases compared with AP. Her serum bilirubin was elevated, and her serum albumin was reduced. This pattern may indicate hepatocellular injury (hepatitis); however, the low albumin level can be attributed to its being a negative acute-phase reactant, and the improvement occurred after the resolution of the liver injury.

A similar pattern of levothyroxine-induced liver injury has been reported [1–3]. Kawakami *et al.* [1] speculated that the complex of levothyroxine as the hapten and liver-related macromolecules in the body of the patient in their case report might have acquired antigenicity, which subsequently resulted in the liver injury. Kang *et al.* [3] interestingly showed that after their patient experienced DILI due to levothyroxine in tablet form, the same was not observed when the patient was commenced on levothyroxine in powder form. This may suggest a casual effect due to the additives contained in these preparations. The mechanisms behind levothyroxine-induced liver injury therefore remain unclear [4]. Among patients in all three previous reports and our patient, none underwent liver biopsy, and in all cases, liver enzymes normalized after cessation of the drug. We opted not to offer liver biopsy. Experts may argue that DIAIH, which can recur at a later stage, cannot be absolutely excluded without a liver biopsy. There is a lack of data comparing these patients with those who have autoimmune hepatitis [5]. Our patient did not have any positive autoimmune antibody tests results and required no immunosuppression, both of which argue against DIAIH, albeit that some patients with DIAIH recover without the need for immunosuppression. Treating our patient’s hypothyroidism with triiodothyronine was effective and did not lead to liver enzyme derangement.

## Conclusion

This case report provides a few learning points. Levothyroxine-induced liver injury is a rare and reversible adverse effect. Clinical vigilance is required when initiating the drug. Triiodothyronine is an appropriate alternative to levothyroxine in treating such cases. Invasive procedures such as liver biopsy were not required to support the diagnosis in our patient’s case. The exact mechanisms of how levothyroxine causes liver injury require further research.

## Data Availability

All the investigation results and reports are available on request.
